# Hypothesis: ultrasonography can document dynamic *in vivo* rouleaux formation due to mobile phone exposure

**DOI:** 10.3389/fcvm.2025.1499499

**Published:** 2025-02-11

**Authors:** Robert R. Brown, Barbara Biebrich

**Affiliations:** 1 Radiology Partners – Phoenix, Mesa, AZ, United States; 2 Environmental Health Trust, Jackson, WY, United States; 3 Department of Radiology, UPMC Hamot, Erie, PA, United States; 4 Pueblo Medical Imaging, Las Vegas, NV, United States

**Keywords:** cellphone, mobile phone, EMFs, radiofrequency radiation, wireless communication, rouleaux, blood viscosity

## Abstract

Carrying a cellphone against the body has become commonplace in our world replete with smartphones. Acute and chronic health effects caused by these devices emitting radiofrequency radiation from multiple antennas have not been well evaluated. In this study, the popliteal vein of a healthy volunteer was imaged with ultrasonography prior to and following the placement of an idle, but active smartphone against her knee for 5 min. Pre-exposure longitudinal sonographic images demonstrate a normal anechoic lumen to the popliteal vein. Images obtained 5 min after direct skin exposure to the smartphone demonstrate a dramatic change in the acoustic appearance of the vessel. The interior of the vessel became coarsely hypoechoic with sluggish flow seen in real-time images, a typical sonographic appearance for rouleaux formation. A follow up examination performed 5 min after the subject walked around yielded continued rouleaux formation in the popliteal vein, albeit less dramatic than that observed immediately post exposure. This revolutionary *in vivo* method to assess radiofrequency radiation induced rouleaux formation should be further pursued in the general population to determine its prevalence and if its occurrence provides a unique biomarker of exposure that may predict morbidity.

## Introduction

Researchers have reported red blood cell (RBC) aggregation, referred to as rouleaux formation, in people who have been recently exposed to electromagnetic fields and radiofrequency radiation. To date, the static technique of live blood cell analysis utilizing dark-field microscopy has been the method of choice to evaluate this phenomenon. Because this *in vitro* analysis may be compromised by artifact from imperfect technique, we sought to produce a novel and innovative approach to this question by devising a noninvasive, *in vivo* method for assessing the presence of rouleaux formation. Diagnostic ultrasound has been the preferred modality for evaluating the blood flow pattern in veins for decades. Although studies are often performed to assess for deep venous thrombosis or venous insufficiency, the presence of rouleaux formation can be readily observed. We hypothesize that ultrasonography provides a simple, non-invasive *in vivo* diagnostic tool to detect the presence of rouleaux formation in individuals following exposure to radiofrequency radiation.

## Method

We performed a series of studies on a 62-year-old asymptomatic healthy female volunteer with no history of allergy, blood disorder, or systemic disease. The volunteer is not on any medication and her only remarkable medical history is having received a pneumococcal vaccine for a lack of pneumococcal antibodies during the previous year. She had no available blood work.

**Figure 1:**
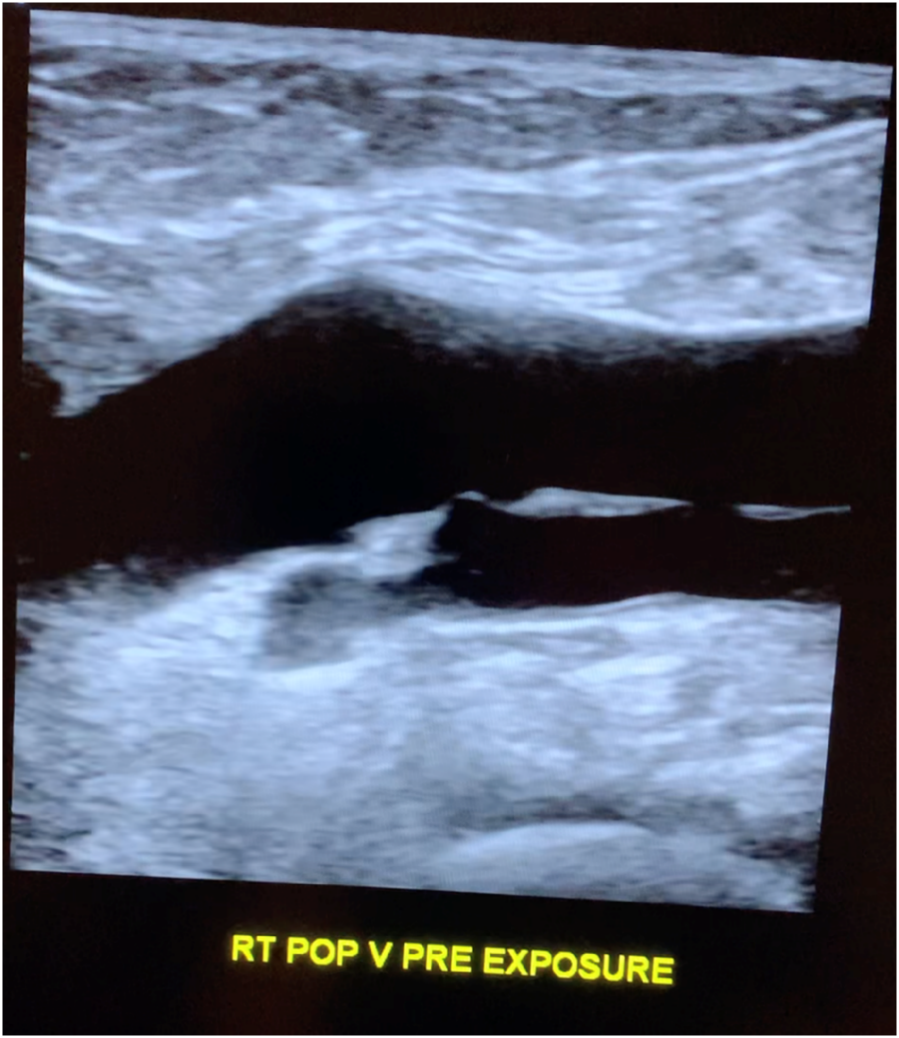
Longitudinal popliteal vein pre-exposure.

The subject was placed on a gurney and draped with her leg exposed. A GE Logic E10 ultrasound machine was utilized with an L2–9 linear probe to image the popliteal fossa. The machine has auto focus and time-gain compensation (TGCs) on a touch screen menu, which can be adjusted by the sonographer to optimize images. A senior ultrasonographer with over 25 years of experience performing vascular ultrasound identified the popliteal vein and obtained cine longitudinal images to confirm the vessel lumen was anechoic (Figure 1). Immediately following, an Apple iPhone XR smartphone operating on the AT&T mobile network was placed on the popliteal fossa for 5 min. The phone's Wi-Fi, Bluetooth, and cellular data antennas were all turned on, but the phone was otherwise inactive and idle. No calls or text messages were received during the 5-min time interval. Note however that even when a phone is not being used to make a call or send a text, devices continually update apps that require uploading and downloading from cellular networks.

**Figure 2:**
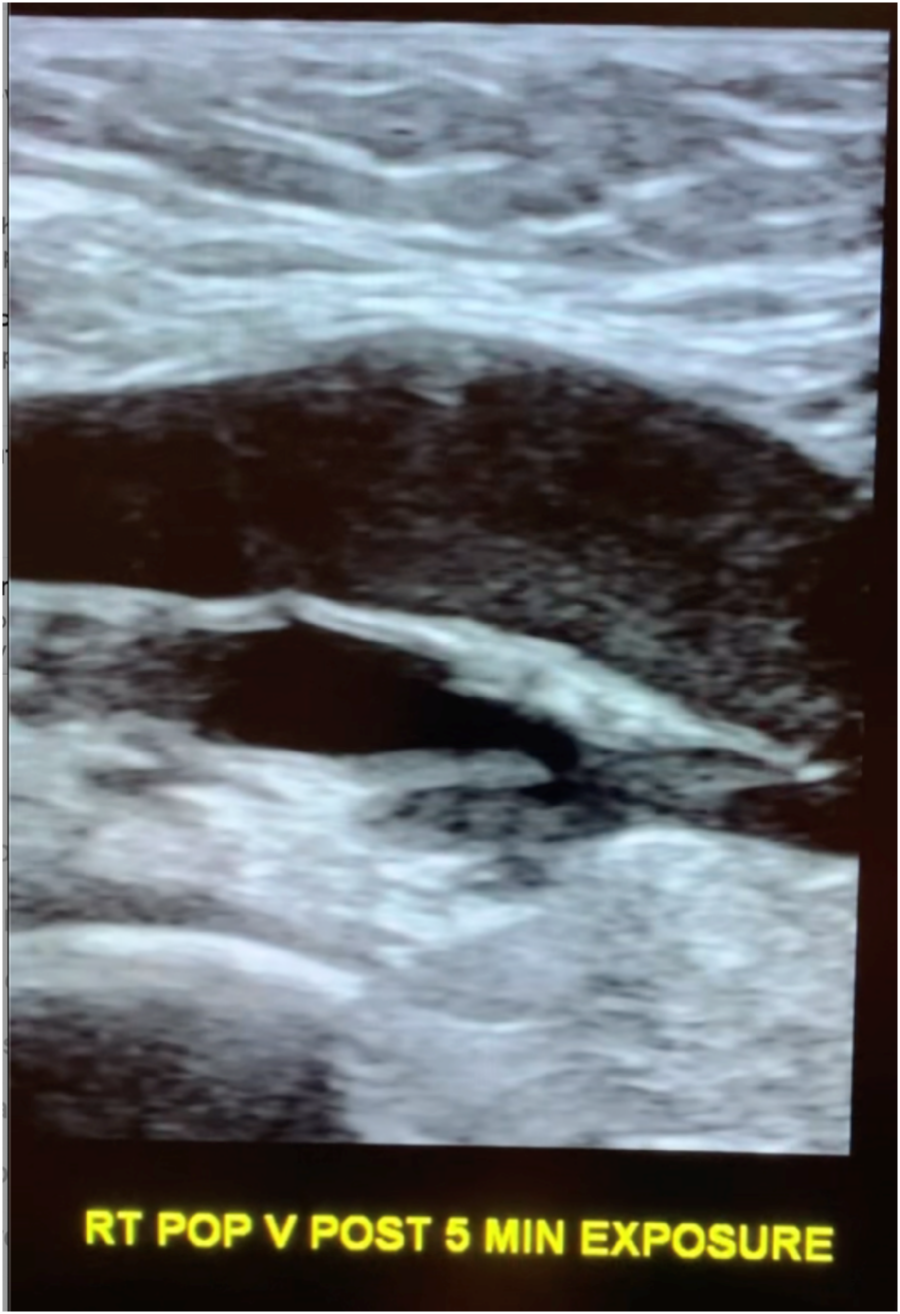
Longitudinal popliteal vein after 5 min of exposure demonstrating rouleaux.

Following exposure, the subject's popliteal vein was reimaged (Figure 2). No changes were made in between the two scans on the ultrasound consol. Specifically, there was no adjustment to the total gain or TGCs that could cause a change in apparent echogenicity of the popliteal structures as compared to pre-exposure images. A post exposure cine loop demonstrates abnormal heterogeneous, predominately hypoechoic material sluggishly moving to and fro within the popliteal vein and nearby tributaries. The sonographic appearance is typical for rouleaux formation, named for the histologic appearance of red blood cells when they are stacked upon one another, resembling a stack of coins. The subject experienced no symptoms.

**Figure 3:**
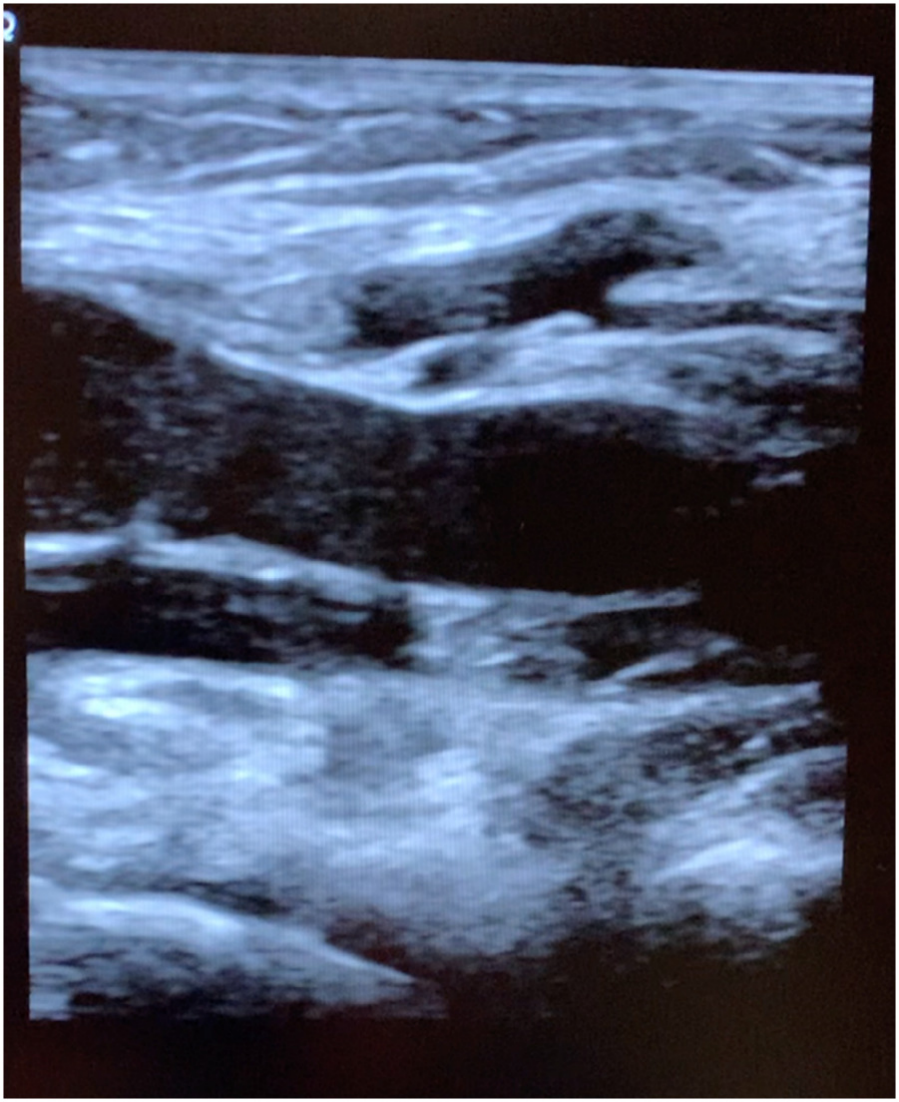
Longitudinal popliteal vein 10 min after exposure demonstrating residual rouleaux.

The subject walked for 5 min after the second scan to see if the rouleaux formation would dissipate with exercise and then reimaged a 3rd time. The final imaging cine loop (10 min after exposure) demonstrate continued rouleaux formation, but the conspicuity of the aggregates had diminished as compared to the immediate post exposure images (Figure 3).

Two months after the initial study was performed, the subject returned to the ultrasound department and was reimaged utilizing the same protocol. Pre-exposure images demonstrated a normal anechoic lumen in the popliteal vein. Images obtained 5 min after cell phone exposure to the popliteal fossa again produced rouleaux formation, confirming reproducibility of the initial observation.

The subject returned 6 weeks later for a third and final assessment. During this imaging session, grey scale and duplex doppler pre-exposure images of the right and left popliteal vein were taken with the subject supine and also while standing. The pre-exposure images demonstrated a normal anechoic lumen to the popliteal veins in both lower extremities. An Apple iPhone 16 plus was then placed against the right popliteal fossa for 5 min. Following, images of the right and left popliteal veins were then obtained with the subject supine and standing. Post exposure images demonstrate rouleaux formation in both lower extremities.

## Discussion

Ultrasound has been the imaging modality of choice to study the extremity veins for decades, replacing the invasive procedure of venography. Imaging is routinely performed to evaluate for the presence or absence of intraluminal clots, i.e., thrombosis. The standard imaging parameters evaluated to diagnose a normal vein are complete luminal compressibility, an anechoic interior, and spontaneous, phasic flow on duplex doppler images. On rare occasion, heterogeneous echogenicity is seen within the lumen and the flow pattern can be observed in real time as an innumerable number of echoes travel through the lumen. The recognition that this sonographic appearance was caused by RBC aggregation or rouleaux formation was first described by Siegel in 1982 (1) and again by Wang et al., in 1992 (2). Rouleaux formation on ultrasound is an uncommon finding, but has been described in patients with infections and inflammatory processes, Waldenström's macroglobulinemia, connective tissue disease, and some forms of cancer, including multiple myeloma, and ischemic injury. Rouleaux can occur from an increase in plasma proteins (3).

Rouleaux formation from exposure to polarized external electromagnetic fields was first reported by Sebastián et al. (4). In 2015, Panagopoulos et al., attributed the increased biological activity of man-made polarized electromagnetic fields to their ability to generate the oscillation of charged molecules and free ions within and surrounding cells, impacting the cell membrane potential and creating an electrochemical imbalance in the cell (5). In this study, because blood chemistry would not change between pre and post exposure images, the aggregation of RBCs into rouleaux is not from a change in concentration of intravascular proteins or other biochemical process, and more likely from polarized electromagnetic fields produced by the cellphone.

Under normal circumstances, erythrocytes have a negative surface charge (zeta-potential), which causes the cells to repel each other from over a distance of 20 nm (6). The zeta-potential is dependent on the ionic strength and dielectric constant of the bulk medium (7). Aggregation occurs when the zeta-potential goes below a critical level. The presence of rouleaux formation on ultrasound indicates these surface charges have weakened and that the cell membrane potential of the RBCs, change upon exposure to EMFs, making the cells adhere to one another.

In 2013, Havas documented rouleaux formation with live blood cell analysis following a 10-min human exposure to a 2.4 GHz cordless phone by utilizing dark field microscopy (8). Although not peer reviewed, Rubik reported red blood cell (RBC) aggregation via dark field microscopy in 10 human subjects who had been exposed to mobile phone radiation for two consecutive 45-min intervals (9). Live blood analysis utilizing dark field microscopy is a static technique criticized as being fraught with potential technical challenges that can lead to false positive results. As such, the findings reported by both researchers have largely been discounted by the scientific community. In comparison, sonography is a useful, non-invasive tool affording dynamic *in vivo* assessment of RBC aggregation and altered flow characteristics without potential technical errors prone to dark field microscopy. There is no risk for damage to the RBCs from blood extraction or manipulation in tubing, tubes or plates. Nor is the time period between extraction and analysis relevant. Furthermore, *in vivo* analysis affords the ability to document dynamic changes in real-time.

The Apple XR iPhone was released to the market in September 2018 and employs several antennas, including a near field communication (NFC) antenna and a 2X2 multiple-input multiple-output (MIMO) antenna. The phone uses long-term evolution (LTE) Advanced technology for telecommunications. These are commonly used by many cellphone vendors and therefore the physiological impact of the phone on the blood is not likely limited to the iPhone XR. Indeed, the third time this study was repeated, the subject was exposed to an Apple iPhone 16 plus. Although the cellphone was inactive during the exposure period, it is well known that frequent, periodic communication “handshaking” via the emission of radiofrequency radiation occurs between the cellphone and cell tower to maintain connectivity with the cellular network while the phone is turned on.

Because tissue perfusion is inversely proportional to blood viscosity, the potential development of rouleaux formation from cellphone exposure is of great concern. Rouleaux formation creates a hypercoagulable state, and may impair oxygen delivery, contributing to tissue ischemia. If the red blood cell aggregation response is indeed systemic, it may have wide reaching multi-systemic effects, including the development or exacerbation of hypertension (10). Morbidity is determined by the patient's underlying health status. Ischemic heart disease, diabetes, prethrombotic states, cancer, peripheral vascular disease, retinopathy, and cerebrovascular insufficiency are among the risk factors that will increase the morbidity associated with the development of rouleaux (11). Although rouleaux is a transient phenomenon, the frequent use of cellphones throughout the day, and potentially other technology commonly found in today's society, may repetitively increase blood viscosity and contribute to micro-occlusions, micro-infarctions, and micro-gangrene. Excessive aggregation of erythrocytes can increase one's susceptibility to develop acute infections, myocardial infarction, and increase one's the risk for deep venous thrombosis (12). As one would expect, if a blood vessel endothelium is damaged or is narrowed by atherosclerotic plaque or other etiology, increased blood viscosity will further compromise flow, reduce the efficiency of gas exchange, and impair oxygenation.

In this study, we exposed the popliteal fossa of the lower extremity to 5 min of radiation from a common iPhone. But we further hypothesize the phenomenon will occur when a phone is placed in a front pocket, and importantly, when held up to the head, affecting the cerebral vasculature in susceptible individuals. Furthermore, the presence of rouleaux in the contralateral extremity as documented during our third study, suggests the red blood cell aggregation becomes a systemic process.

First-level hypotheses can serve an important role for the subsequent identification of exposure-disease relationships. Conclusions based on this study however are limited as we only assessed one individual at three points in time. Because ultrasound is readily available, future studies can be performed on larger populations to assess the incidence of this occurrence and to better define at which exposure time rouleaux formation becomes clinically observable, and which frequencies, modulation patterns, and power densities of radiofrequency radiation cause this phenomenon to occur. Although our subject experienced no symptoms and was unaware and surprised that her blood had become aggregated, it should be interesting to determine if transient symptoms, such as fatigue, develop in others when they go into rouleaux formation from EMF exposure.

In summary, we present a subject in whom 5 min of exposure to radiofrequency radiation emitted by a smartphone causes abnormal erythrocyte aggregation “rouleaux formation” *in vivo*. This abnormality is associated with sluggish venous flow, as documented on diagnostic ultrasound obtained in real time. Although rouleaux formation is recognized as a transient phenomenon, we hypothesize that habitual cellphone usage, common in the population, would re-expose individuals over and over again to this abnormal hematologic state. Chronic, long-term exposure to radiofrequency radiation may therefore lead to recurrent, chronic RBC aggregation and increased blood viscosity, potentially causing significant morbidity in certain patient populations, particularly diabetics and those with hypertension, ischemic heart disease, cerebrovascular insufficiency, prethrombotic states, and peripheral vascular disease. Recognizing the potential for red blood cell aggregation from radiofrequency radiation to occur in the general population is crucial. Further studies are needed to assess the incidence of this occurrence, in addition to defining which power densities and frequencies put individuals at risk.

## Data Availability

The original contributions presented in the study are included in the article/Supplementary Material, further inquiries can be directed to the corresponding author.
