# Correction: Hypothesis: ultrasonography can document dynamic *in-vivo* rouleaux formation due to mobile phone exposure

**DOI:** 10.3389/fcvm.2026.1724889

**Published:** 2026-02-19

**Authors:** Robert R. Brown, Barbara Biebrich

**Affiliations:** 1 Radiology Partners – Phoenix, Mesa, AZ, United States; 2 Environmental Health Trust, Jackson, WY, United States; 3 Department of Radiology, UPMC Hamot, Erie, PA, United States; 4 Pueblo Medical Imaging, Las Vegas, NV, United States

**Keywords:** cellphone, mobile phone, EMFs, radiofrequency radiation, wireless communication, rouleaux, blood viscosity

The acknowledgements was erroneously given as “The authors would like to thank Dr. Devra Davis and Dr. Kent Chamberlin for their encouragement, incite, and expert assistance in obtaining IRB approval.” The correct acknowledgements is “The authors would like to thank Dr. Devra Davis and Dr. Kent Chamberlin for sharing their encouragement and expertise.”

The ethics statement was erroneously given as “The studies involving humans were approved by University of New Hampshire Research Integrity Services. The studies were conducted in accordance with the local legislation and institutional requirements. The participant(s) provided their written informed consent to participate in this study. Written informed consent was obtained from the individual(s) for the publication of any potentially identifiable images or data included in this article.” The correct ethics statement is “The study was conducted on and with the written informed consent of author BB, without ethical approval having been sought or obtained. Approval was granted on January 8, 2025, by the University of New Hampshire Research Integrity Services, for a new study planned to prospectively involve up to 40 participants, but did not include the participant/author mentioned in the current study.”

The conflict-of-interest statement was erroneously given as “The authors declare that the research was conducted in the absence of any commercial or financial relationships that could be construed as a potential conflict of interest.” The correct conflict of interest statement is “The authors declare that the research was conducted in the absence of any commercial or financial relationships that could be construed as a potential conflict of interest. At the time of publication, the handling editor was a board member of the Environmental Health Trust, and the first author was an executive committee member of that same non-profit organization.”

The original version of this article has been updated.

